# Low genetic diversity and functional constraint in loci encoding *Plasmodium vivax* P12 and P38 proteins in the Colombian population

**DOI:** 10.1186/1475-2875-13-58

**Published:** 2014-02-18

**Authors:** Johanna Forero-Rodríguez, Diego Garzón-Ospina, Manuel A Patarroyo

**Affiliations:** 1 Molecular Biology and Immunology Department, Fundación Instituto de Inmunología de Colombia (FIDIC), Carrera 50 No. 26-20, Bogotá, DC, Colombia; 2 Microbiology postgraduate programme, Universidad Nacional de Colombia, Bogotá, DC, Colombia; 3 School of Medicine and Health Sciences, Universidad del Rosario, Bogotá, DC, Colombia

**Keywords:** 6-Cys, *pv12*, *pv38*, s48/45 domain, Functional constraint, *Plasmodium vivax*, Genetic diversity, Anti-malarial vaccine

## Abstract

**Background:**

*Plasmodium vivax* is one of the five species causing malaria in human beings, affecting around 391 million people annually. The development of an anti-malarial vaccine has been proposed as an alternative for controlling this disease. However, its development has been hampered by allele-specific responses produced by the high genetic diversity shown by some parasite antigens. Evaluating these antigens’ genetic diversity is thus essential when designing a completely effective vaccine.

**Methods:**

The gene sequences of *Plasmodium vivax p12* (*pv12*) and *p38* (*pv38*), obtained from field isolates in Colombia, were used for evaluating haplotype polymorphism and distribution by population genetics analysis. The evolutionary forces generating the variation pattern so observed were also determined.

**Results:**

Both *pv12* and *pv38* were shown to have low genetic diversity. The neutral model for *pv12* could not be discarded, whilst polymorphism in *pv38* was maintained by balanced selection restricted to the gene’s 5′ region. Both encoded proteins seemed to have functional/structural constraints due to the presence of s48/45 domains, which were seen to be highly conserved.

**Conclusions:**

Due to the role that malaria parasite P12 and P38 proteins seem to play during invasion in *Plasmodium* species, added to the Pv12 and Pv38 antigenic characteristics and the low genetic diversity observed, these proteins might be good candidates to be evaluated in the design of a multistage/multi-antigen vaccine.

## Background

Malaria is a disease caused by protozoan parasites from the *Plasmodium* genus*,* five of which cause the disease in human beings (*Plasmodium falciparum, Plasmodium vivax, Plasmodium ovale, Plasmodium malariae and Plasmodium knowlesi*) [1,2]. This parasite is transmitted by the bite of an infected *Anopheles* female mosquito. Around 3.3 billon people are at risk of malaria annually, mainly in tropical and subtropical areas of the world, children aged less than five years and pregnant women being the most vulnerable [3]. *Plasmodium falciparum* is responsible for the disease’s most lethal form, being predominantly found on the African continent whilst *P. vivax* is widely distributed around the world. Even though it has been thought that infection caused by the latter species was benign, recent studies have shown that *P. vivax* can cause clinical complications [4]. It has been found that 2,488 million people are at risk of becoming infected by *P. vivax* on the continents of Asia and America, 132 to 391 million cases occurring annually [5].

In spite of control strategies having been introduced in different countries, malaria continues to be a public health problem due to the parasite’s resistance to anti-malarial treatments [6] and the vector’s resistance to insecticides [7], among other causes. More effective measures have thus to be implemented for controlling such disease, including the development of an anti-malarial vaccine.

Several antigens have been characterized as promising candidates for inclusion in a vaccine [8,9], however, the genetic diversity of some of them [10-18] has hampered the development of such vaccine [19,20] as these genetic variations provoke allele-specific responses [21,22] making them become a mechanism for evading the immune system [23]. It has been necessary to focus vaccine development on conserved domains or antigens to avoid such responses [24], since these regions could have functional constraint and have had slower evolution [25].

Developing a multi-antigen vaccine against the parasite’s blood stage has been focused on blocking all host-pathogen interactions to stop merozoite entry to red blood cells (RBC) [26]. A group of proteins anchored to the membrane via glycosylphosphatidylinositol (GPI) has been identified in *P. falciparum*, predominantly located in detergent-resistant membrane (DRM) domains [27,28]; they have been implicated in the parasite’s initial interaction with RBC [29-33] and some have been considered as being candidates for being included in a vaccine [34,35]. One group of proteins belonging to the 6-cystein (6-Cys) family is particularly noteworthy among these DRMs (i.e., Pf12, Pf38, Pf41 and Pf92) as they have been characterized by having s48/45 domains (ID in PFAM: PF07422). Members of this family are expressed during different parasite stages [28,36] and some of them (e.g., Pf48/45, Pf230) have been considered as vaccine candidates for the sexual stage [36,37].

Pf12 and Pf38 are expressed during late stages of the intra-erythrocyte cycle, each having two high binding peptides, suggesting an active role during invasion of RBC [30]. Orthologous genes encoding these proteins have been characterized recently in *P. vivax*[38,39]. Both proteins have a signal peptide, a GPI anchor sequence and have been associated with DRMs [38,39]. Pv12 has two s48/45 domains [39] whilst Pv38 has a single domain located towards the C-terminal end [38]*.* These proteins have been shown to be antigenic [38-40], suggesting that they are exposed to the immune system, probably during *P. vivax* invasion of RBC.

The present study involved a population genetics analysis for evaluating the genetic diversity of *pv12* and *pv38* loci and the evolutionary processes generating this variation pattern; the results revealed these antigens’ low genetic diversity in the Colombian population, possibly due to functional/structural constraints in s48/45 domains. Since the proteins encoded by these genes share structural characteristics with other vaccine candidates, added to the fact that Pv12 and Pv38 are targets for the immune response [38-40] and have conserved domains, they should be considered when designing a multistage/multi-antigen anti-malarial vaccine.

## Methods

### Ethics statement

The parasitized DNA used in this study was extracted from total blood collected from different Colombian areas (Antioquia, Atlántico, Bogotá, Caquetá, Cordoba, Chocó, Guainía, Guaviare, Magdalena, Meta, Nariño, and Tolima) from 2007 to 2010. All *P. vivax*-infected patients who provided blood samples were notified about the object of the study and signed an informed consent form if they agreed to participate. All procedures involved in taking blood samples were approved by Fundación Instituto de Inmunología de Colombia (FIDIC) ethics committee.

### Parasitized DNA presence and integrity

Parasitized DNA presence and integrity in 100 samples stored at -20°C (2007-2010) at FIDIC (from different areas of Colombia) were evaluated by *18S* ribosomal RNA gene amplification using specific primers for *P. vivax* (SSU-F 5′-ATGAACGAGATCTTAACCTGC-3′ and SSU-R 5′-CATCACGATATGTA5TGATAAAGATTACC-3′) in a touchdown PCR [41]. The reaction contained: 1x Mango Taq reaction buffer (Bioline), 2.5 mM MgCl_2_, 0.25 mM dNTPs, 0.5 mM of each primer, 0.1 U Mango Taq DNA polymerase (Bioline) and 10-40 ng gDNA in 10 mL final volume. The PCR thermal profile was: one initial denaturing cycle at 95°C (5 min), followed by ten cycles at 95°C (20 sec), annealing at 65°C (30 sec) and an extension step at 72°C (45 sec). Annealing temperature was reduced by 1°C in each cycle until reaching 55°C; 35 additional cycles were run at this temperature followed by a final extension cycle at 72°C (10 min). PCR products were visualized by electrophoresis on 1.5% agarose gel in 1× TAE, using 1 μL SYBR-Safe (Invitrogen).

### Identifying infection caused by single *Plasmodium vivax* strain

Infection by the single *P. vivax* strain was identified by PCR-RFLP of the *pvmsp-1* polymorphic marker. The *pvmsp-1* gene fragment 2 (blocks 6, 7 and 8) was amplified using direct 5′-AAAATCGAGAGCATGATCGCCACTGAGAAG-3′ and reverse 5′-AGCTTGTACTTTCCATAGTGGTCCAG-3′ primers [42]. The amplified fragments were digested with Alu I and Mnl I restriction enzymes, as described elsewhere [42]. The products were visualized by electrophoresis on 3% agarose gel in 1× TAE, using 1 μL SYBR-Safe (Invitrogen).

### PCR amplification of *pv12* and *pv38* genes

A set of primers was designed for amplifying each of the genes based on Sal-I reference strain sequences (accession numbers in PlasmoDB: PVX_113775 for *pv12* and PVX_097960 for *pv38*). The following primers were used: for *pv12*, *pv12-*direct 5′-GTACCGCTTAACACCGC-3′ and *pv12-*reverse 5′-GCACTACATTATAAAGAAAAGGACC-3′ and for *pv38*, *pv38-*direct 5′-CGCTTCTTTCACCGCTTC-3′ and *pv38-*reverse 5′-CACACATTAACGCTGCTTCG-3′. The PCR reaction mixture contained 10 mM Tris HCL, 50 mM KCl (GeneAmp 10× PCR Buffer II [Applied Biosystems]), 1.5 mM MgCl_2_, 0.2 mM of each dNTP, 0.5 μM of each primer, 0.76 U Amplitaq Gold DNA polymerase (Applied Biosystems) and 10-40 ng gDNA in a 50 μL final volume. The PCR thermal profile was as follows: one cycle at 95°C (7 min), 40 cycles at 95°C (20 sec), 56°C (30 sec), 72°C (1 min) and a final extension cycle at 72°C (10 min). PCR products were purified using a commercial UltraClean PCR Clean-up kit (MO BIO). The purified PCR products were sequenced in both directions with the amplification primers using the BigDye method with capillary electrophoresis, using ABI-3730 XL (MACROGEN, Seoul, South Korea). Two independent PCR products were sequenced to ensure that errors were ruled out.

### Analysing genetic diversity

The electropherograms obtained by sequencing were analysed and forward and reverse sequences were assembled using CLC Main workbench software v.5 (CLC bio, Cambridge, MA, USA). The *pv12* and *pv38* genes were analysed and compared to reference sequences obtained from several sequencing projects [43,44] (accession numbers, *pv12*: XM_001616094.1, AFBK01001496.1, AFNI01000939.1, AFMK01001167.1 and AFNJ01001458.1; *pv38*: XM_001613202.1, AFNI01000834.1, AFNJ01000090.1, AFMK01001057.1 and AFBK01001340.1) or those reported in the GenBank database (accession numbers for *pv12*: GU476521.1; and for *pv38*: JF427569.1 and JF427570.1). Gene Runner software was used for translating the sequences for deducing the amino acid sequences. These sequences were then aligned using the MUSCLE algorithm [45], and manually edited. Amino acid alignment was then used for inferring DNA using PAL2NAL software [46].

DnaSP software (v.5) [47] was used for evaluating intrapopulation genetic polymorphism by calculating: the number of polymorphic segregating sites (Ss), the number of singleton sites (s), the number of parsimony-informative sites (Ps), the number of haplotypes (H), haplotype diversity (Hd, which was multiplied by (n-1)/n according to Depaulis and Veuille [47,48]), the Watterson estimator (θw) and nucleotide diversity per site (π). DNA sequence variation was calculated using the sequences obtained from the aforementioned databases, plus the Colombian ones (worldwide isolates, global diversity) and just those obtained for the Colombian population (local diversity). The frequency for each Colombian haplotype was also estimated by count and year.

Two test families were used for evaluating the neutral molecular evolution model for the Colombian population: (1) frequency spectrum test, and (2) haplotype test. The former involved calculating Tajima’s D statistics [49], Fu and Li’s D* and F* [50] and Fay and Wu’s H statistic [51]. Tajima’s D statistic compares the difference between segregating sites and the average of nucleotide differences between two randomly taken sequences. Fu and Li’s D* statistic takes the difference between the number of singleton sites and the total of mutations, whilst F* takes the difference between the number of singleton sites and the average of nucleotide differences between two randomly taken sequences. Fay and Wu’s H statistic is based on the difference of the average number of nucleotide differences between pairs of sequences and the frequency of the derived variants. Fu’s Fs statistic [52], K-test and H-test [48] are tests for calculating haplotype distribution. The Fs statistic compares the number of haplotypes observed to the expected number of haplotypes in a random sample. K-test and H-test [48] are based on haplotype number and haplotype diversity, respectively; these statistics are conditioned by sample size (n) and the number of segregating sites (Ss). Test significance was determined by coalescence simulations using DnaSP (v.5) [47] and ALLELIX software (kindly supplied by Dr Sylvain Mousset). Sites having gaps were not taken into account in any of the tests performed.

The effect of natural selection was evaluated regarding intra and interspecies; the average number of non-synonymous substitutions per non-synonymous site (d_N_) and the average number of synonymous substitutions per synonymous site (d_S_) were calculated for the former by using the modified Nei-Gojobori method [53]. The significant differences between the above were determined by using Fisher’s exact test (suitable for d_N_ and d_S_ < 10) and codon-based Z-test incorporated in MEGA software (v.5) [54]. Differences between d_N_ and d_S_ per site were calculated by using SLAC, FEL, REL [55], IFEL [56], MEME [57], and FUBAR [58] methods. The average number of non-synonymous divergence substitutions per non-synonymous site (K_N_) and the average number of synonymous divergence substitutions per synonymous site (K_S_) were calculated using the modified Nei-Gojobori method [53], with Jukes-Cantor correction [59], to infer natural selection signals which may have prevailed during malarial parasite evolutionary history (interspecies; using *Plasmodium cynomolgi* (accession number BAEJ01001076.1) and *P. knowlesi* (accession number NC_011912.1) orthologous sequences). The significant differences between K_N_ and K_S_ were determined by using a codon-based Z-test incorporated in MEGA software (v.5) [54]. The McDonald-Kreitman test [60] was also calculated; this is based on a comparison of intraspecific polymorphism to interspecific divergence (using *Plasmodium cynomolgi* (accession number BAEJ01001076.1) and *P. knowlesi* (accession number NC_011912.1) orthologous sequences). This test involved using a web server [61], which takes Jukes-Cantor divergence correction into account [59]. All the above tests were calculated using the sequences obtained from the databases plus the Colombian ones and just those obtained for the Colombian population.

Z_nS_[62] and ZZ [63] statistics were calculated for evaluating the influence of linkage disequilibrium (LD) and intragenic recombination, respectively. The minimum number of recombination (Rm) events was also calculated; this included calculating effective population size and the probability of recombination between adjacent nucleotides per generation [64]. Additionally, the GARD method [65] available at the Datamonkey web server [66] was performed. These tests were performed using the sequences obtained from the Colombian population.

## Results and discussion

### The presence of genomic DNA (gDNA) and identification of single *Plasmodium vivax* strain infection

An *18S* subunit rRNA gene fragment was amplified from 100 samples of *P. vivax* collected from different areas of Colombia and stored from 2007 to 2010. Seventy-seven samples revealed an amplicon at the expected size, indicating the presence of *P. vivax* gDNA. A region of the *pvmsp-1* gene was then amplified and digested with restriction enzymes, showing that seven of the 77 samples proving positive for *P. vivax* had multiple infections. Only 70 samples were thus considered for later analysis. Due to the low number of samples collected from some areas, they were grouped according to geographical localisation and epidemiological conditions (South-west: Chocó, Nariño; South-east: Caquetá, Guainía, Guaviare, Meta; Midwest: Bogota, Tolima; North-west: Atlántico, Antioquia Cordoba, Magdalena).

### Genetic diversity in *pv12*

Seventy samples amplified a 1,200 base pair (bp) fragment corresponding to the *pv12* gene (South-west n = 6; South-east: n = 20; Midwest: n = 8; North-west: n =36). These amplicons were purified and sequenced; the sequences were then analysed, compared to different reference sequences obtained from various sequencing projects [43,44] and those having a different haplotype were deposited in the GenBank database (accession numbers KF667328 and KF667329).

**Figure 1:**
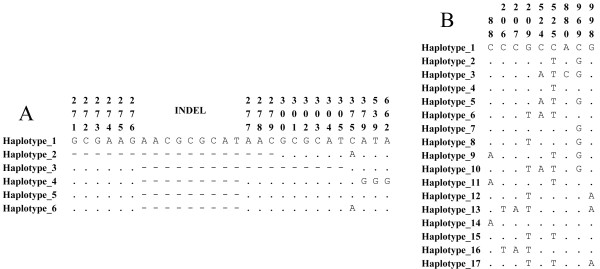
***pv12 *and *pv38 *haplotype alignment. A.** Alignment of the six haplotypes found in the *pv12* gene. Haplotypes 2, 3, 5 and 6 were found in the Colombian population. **B.** Alignment of the 17 haplotypes found in *pv38*, 14 of them found in the Colombian population (haplotypes 1, 5, 6, 7, 8, 9, 10, 11, 12, 13, 14, 15, 16, and 17). The dots indicate nucleotide identity and dashes indicate nucleotide absence. Numbering is based on the Sal-I reference sequence.

Four single nucleotide polymorphisms (SNP) were observed throughout the *pv12* gene sequence (Figure 1A) located in positions 375 (N125K), 379 (T127A), 539 (L180W) and 662 (N221S). Only one SNP (nucleotide 375) was found in the Colombian population. A repeat region was observed; it was formed by previously reported amino acids N[A/V][H/Q] [39], in which an insertion was observed in the North Korean sequence (Figure 1A, haplotype 1) and deletions in the Colombian sequences (Figure 1A, haplotypes 2 and 3).

**Figure 2:**
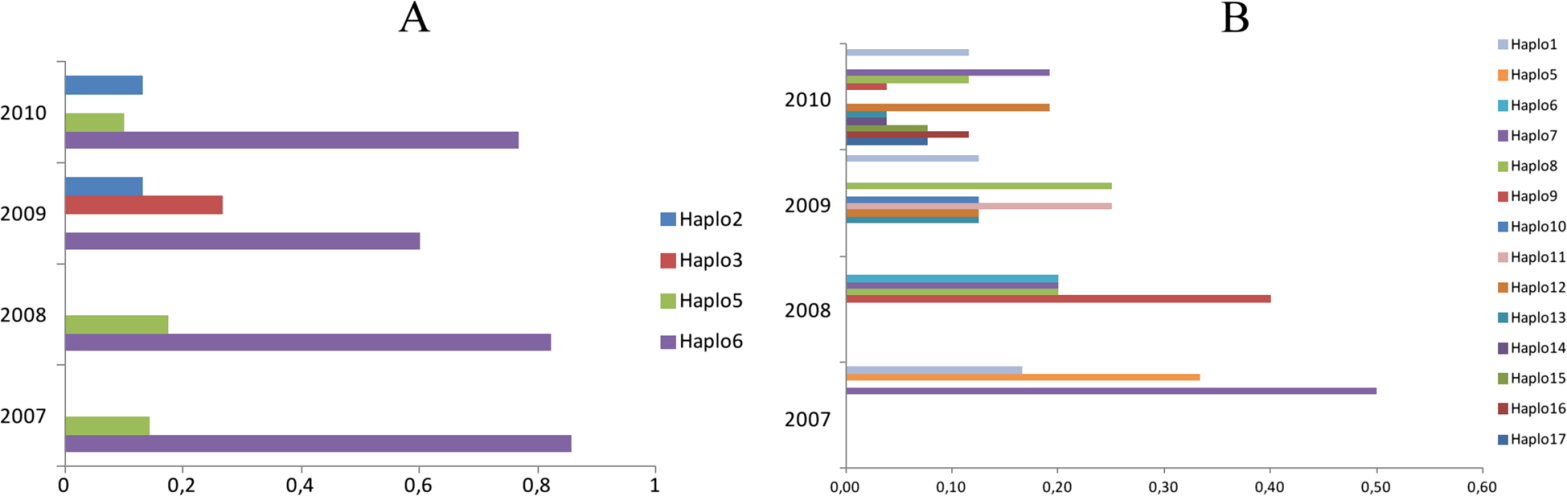
**Frequency of the *pv12 *and *pv38 *haplotypes present in the Colombian population. A.** Frequency per year for the four *pv12* haplotypes found in the Colombian population: n = 9 in 2007, n = 17 in 2008, n = 15 in 2009 and n = 29 in 2010. **B.** Frequency per year for the 14 *pv38* haplotypes found in the Colombian population. n = 6 in 2007, n = 6 in 2008, n = 8 in 2009 and n = 26 in 2010.

Six haplotypes were found in *pv12* (Figure 1A and Table 1) around the world, four of which are present in Colombia at 8.7, 5.8, 10.1, and 75.4% frequency for haplotypes 2, 3, 5 and 6, respectively. Haplotypes 2, 5 and 6 were present in the different Colombian locations (Additional file 1), haplotype 6 being the most predominant per year (2007 n = 9; 2008 n = 17; 2009 n = 15; 2010 n = 29) and per location, having higher than 70% frequency (Figure 2A and Additional file 1). The remaining haplotypes were absent or had low frequency (Figure 2A and Additional file 1). Interestingly, haplotype 3 was present in Colombia during 2009 but absent in the other years studied (Figure 2A). The percentage of samples from the South-east area (some of them presenting haplotype 3) was greater than for other years, suggesting that haplotype 3 was restricted to a particular geographical area (Additional file 1) and/or that this had very low frequency in different Colombian subpopulations. Haplotype 2 was absent from 2007 to 2008 but present between 2009 and 2010 (Figure 2A); differently to haplotype 3, this haplotype was present everywhere, except in the South-west location (Additional file 1). This appeared to be consistent with previous studies which have reported numerous private haplotypes in American *Plasmodium vivax* populations [67]. These results suggested that the Colombian population had one predominant *pv12* haplotype and several low frequency alleles, which are geographically isolated or were not detected during some periods of time. Since *P. vivax* populations within countries seem to be strongly structured [67], new *pv12* haplotypes could appear in other parasite populations.

**Table 1:** **Estimators for *pv12 *** **and *pv38 *** **global and local genetic diversity**

**n**	**Gene**	**Sites**	**Ss**	**S**	**Ps**	**H**	**θw (sd)**	**π (sd)**
**Worldwide isolates**								
76	***pv12***	927	4	3	1	6	0.0009 (0.0005)	0.0004 (0.0001)
53	***pv38***	1,035	9	1	8	17	0.0019 (0.0006)	0.0026 (0.0002)
**Colombian population**								
70	***pv12***	1,047	1	0	1	4	0.0002 (0.0002)	0.0003 (0.0001)
46	***pv38***	1,062	8	0	8	14	0.0017 (0.0006)	0.0024 (0.0002)

Estimators of genetic diversity were calculated using the sequences obtained from databases plus the Colombian ones (worldwide isolates, global diversity) and just those obtained for the Colombian population (Colombian population, local diversity). n: number of isolates, sites: total of sites analysed excluding gaps, Ss: number of segregating sites, S: number of singleton sites, Ps: number of informative-parsimonious sites, H: number of haplotypes, θ_w_: Watterson estimator, π: nucleotide diversity per site. sd: standard deviation.

This gene had 0.0004 ± 0.0001 global nucleotide diversity (π) and 0.0003 ± 0.0001 for the Colombian population (Table 1). This value was about 2.5 times less than that reported for its orthologue in *P. falciparum* (π = 0.001) [68]; however, both values were low when compared to other membrane proteins [10-14,17], suggesting that this gene is highly conserved in different *Plasmodium* species. This value places *pv12* among the most conserved antigen-encoding genes characterized to date in *P. vivax*.

### Mutations in *pv12* appear to be selectively neutral

Several tests for evaluating the hypothesis that mutations in *pv12* are neutral were performed. No significant values were found for the Tajima, Fu and Li, Fay and Wu or Fu tests (Table 2); likewise, the Colombian population’s number of haplotypes (4) and haplotype diversity (0.406 ± 0.07) (Table 2) were as expected under neutrality according to the K-test and H-test. Since neutrality could not be ruled out, the mutations or haplotypes found in *pv12* could have been randomly fixed; this might explain the possible geographical isolation of haplotype 3, since different alleles could have become fixed in different populations according to the neutral hypothesis. Alternatively, the geographical isolation of haplotype 3 could have resulted from the structured *P. vivax* population in America, where haplotypes may have diversified *in situ*.

**Table 2:** ***pv12 *** **and *pv38 *** **neutrality, linkage disequilibrium and recombination tests for the Colombian population**

**N**	**Gene**	**Tajima**	**Fu and Li**		**Fay and Wu’s H**	**Fu’s Fs**	**K-test**	**H-test (sd)**	**Z**_**ns**_	**ZZ**	**RM**
		**D**	D*	F*							
70	***pv12***	0.365	0.516	0.548	0.000	0.902	4	0.406 (0.07)	ND	ND	0
46	***pv38***	1.147	1.304	1.473	−1.275	−4.451	14*	0.890 (0.02)*	0.107	0.125	2

n: number of isolates.

*: p < 0.05.

ND: not determined.

sd: standard deviation.

### Natural selection in *pv12*

The gene was split into two regions: region A, nucleotides 1-546 (amino acids 1-182 including one s48/45 domain) and region B, nucleotides 547-1,095 (amino acids 183-365 including the other s48/45 domain). Synonymous substitution per synonymous site (d_S_) and non-synonymous substitution per non-synonymous site rates (d_N_) were calculated using the gene’s total length to evaluate whether natural selection had any effect on *pv12* evolution. Full length gene and split regions had non-significant values (Table 3); likewise, when d_N_ and d_S_ were estimated for s48/45 domains, no significant values were observed (Additional file 2), contrary to that suggested for *pf12,* where purifying selection action has been reported [69]. The Datamonkey server was used for calculating d_N_ and d_S_ rates for each codon; no selected sites were found, indicating (once more) that the gene did not appear to deviate from neutrality.

**Table 3:** **Synonymous substitution per synonymous site rate (d**_**S**_**) and non-synonymous substitution per non-synonymous site rate (d**_**N**_**) for *pv12 *** **and *pv38 *** **genes**

**n**	**Gene**	**Region A**		**Region B**		**Full length gene**	
		**d**_**S **_**(se)**	**d**_**N **_**(se)**	**d**_**S **_**(se)**	**d**_**N **_**(se)**	**d**_**S **_**(se)**	**d**_**N **_**(se)**
**Worldwide isolates**							
76	***pv12***	0.000 (0.000)	0.001 (0.001)	0.000 (0.000)	0.000 (0.000)	0.000 (0.000)	0.001 (0.000)
53	***pv38***	0.001 (0.001)	0.003 (0.002)	0.006 (0.004)	0.001 (0.001)	0.004 (0.002)	0.002 (0.001)
**Colombian population**							
70	***pv12***	0.000 (0.000)	0.001 (0.001)	0.000 (0.000)	0.000 (0.000)	0.000 (0.000)	0.000 (0.000)
46	***pv38***	0.001 (0.001)	0.003 (0.002)	0.005 (0.004)	0.001 (0.001)	0.004 (0.002)	0.002 (0.001)

d_N_ and d_S_ rates were estimated by using sequences obtained from databases together with Colombian ones (worldwide isolates) and just with those obtained in the Colombian population. n: number of isolates. *pv12*: region A, nucleotides 1-546 and region B, nucleotides 547-1,095. *pv38*: region A, nucleotides 1-459 and region B, nucleotides 460-1,065. se: standard error. No statistically significant differences were found.

**Figure 3:**
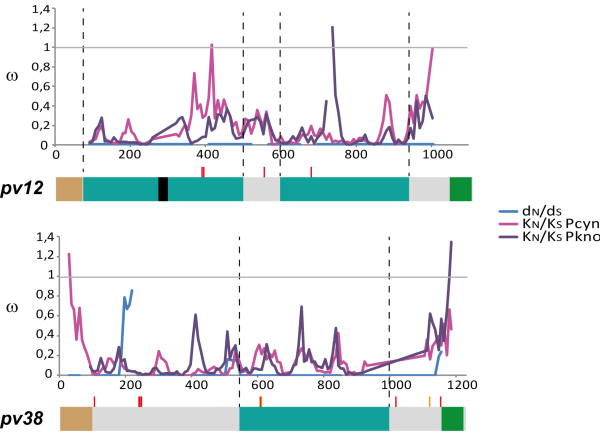
**Sliding window analysis for ω rates.** The ω (d_N_/d_S_) values for *Plasmodium vivax p12* and *p38* are shown in blue, whereas the divergence (ω: K_N_/K_S_) between *Plasmodium vivax* and *Plasmodium cynomolgi* (Pcyn) and *Plasmodium vivax* and *Plasmodium knowlesi* (Pkno) is displayed in magenta and purple, respectively. A gene diagram is shown below the sliding window. Regions encoding signal peptides (brown), GPI anchors (green), s48/45 domains (dark cyan) as well as the N[A/V][H/Q] repeat (black) are indicated. Non-synonymous (red) and synonymous (orange) substitutions are shown with vertical lines above each gene.

However, assessing how natural selection acts on low genetic diversity antigens is not easy [70]; the fact that *Plasmodium vivax* shares its most recent common ancestor with parasites infecting primates (e.g. *P. cynomolgi* and *P. knowlesi*) led to inferring patterns which may have prevailed during their evolutionary history [70,71]. When synonymous divergence substitution per synonymous site (K_S_) and non-synonymous divergence substitution per non-synonymous site (K_N_) rates were calculated, a significantly higher K_S_ than K_N_ was found (Table 4). Moreover, a sliding window for ω (d_N_/d_S_ and/or K_N_/K_S_) revealed < 1 values throughout the gene (Figure 3), which could have been a consequence of negative selection. Moreover, significant values were observed when the McDonald-Kreitman (MK) test was used for comparing intraspecific polymorphism and interspecific divergence (using all the haplotypes found for this gene): P_N_/P_S_ > D_N_/D_S_ (Table 5), revealing (similar to K_S_ rates) a large accumulation of synonymous substitutions between species, which could be interpreted as negative selection. Such accumulation of interspecies synonymous substitutions suggested that evolution tried to maintain protein structure by eliminating all deleterious mutations. However, when the MK test was done with haplotypes found in Colombia (and in spite of the accumulation of synonymous substitutions between species), no significant values were observed in this population (Table 5). Although Pv12 is exposed to the immune system [39,40], it had a high level of conservation. This pattern could have been because *pv12* had diverged by negative selection, due to a possible functional/structural constraint imposed by the presence of s48/45 domains [72] which seem to play an important role during host cell recognition [30,69,72].

**Table 4:** **Synonymous divergence substitution per synonymous site (K**_**S**_**) rate and non-synonymous divergence substitution per non-synonymous site (K**_**N**_**) rate**

***P. vivax/P. Cynomolgi***							
**n**	**Gene**	**s48/45 domain in region A**		**s48/45 domain in region B**		**Full-length gene**	
		**K**_**S **_**(se)**	**K**_**N **_**(se)**	**K**_**S **_**(se)**	**K**_**N **_**(se)**	**K**_**S **_**(se)**	**K**_**N **_**(se)**
**Worldwide isolates**							
78	***pv12***	0.016 (0.003)†	0.005 (0.002)	0.019 (0.004)†	0.003 (0.001)	0.016 (0.002)*	0.004 (0.001)
54	***pv38***	-		0.030 (0.007)†	0.005 (0.001)	0.031 (0.004)*	0.007 (0.001)
**Colombian isolates**							
71	***pv12***	0.018 (0.003)†	0.005 (0.001)	0.021 (0.004)†	0.003 (0.001)	0.016 (0.002)*	0.005 (0.001)
47	***pv38***	-		0.033 (0.007)†	0.006 (0.001)	0.033 (0.004)*	0.008 (0.001)
***P. vivax/P. knowlesi***							
**n**	**Gene**	**s48/45 domain in region A**		**s48/45 domain in region B**		**Full-length gene**	
		**K**_**S **_**(se)**	**K**_**N **_**(se)**	**K**_**S **_**(se)**	**K**_**N **_**(se)**	**K**_**S **_**(se)**	**K**_**N **_**(se)**
**Worldwide isolates**							
78	***pv12***	0.025 (0.005)†	0.006 (0.002)	0.020 (0.004)†	0.003 (0.001)	0.022 (0.003)*	0.005 (0.001)
54	***pv38***	-		0.028 (0.006)†	0.005 (0.001)	0.034 (0.004)*	0.007 (0.001)
**Colombian isolates**							
71	***pv12***	0.027 (0.006)†	0.006 (0.001)	0.022 (0.005)†	0.003 (0.001)	0.023 (0.002)*	0.005 (0.001)
47	***pv38***	-		0.031 (0.007)†	0.005 (0.001)	0.038 (0.005)*	0.008 (0.001)

K_N_ and K_S_ rates were estimated by using sequences obtained from databases (worldwide isolates) together with Colombian ones, and just with those obtained in the Colombian population. n: number of isolates. *pv12* s48/45 domain in region A: nucleotides 82-471; *pv12* s48/45 domain in region B: nucleotides 589-906; *pv38* s48/45 domain in region B: nucleotides 481-852; -: There are no s48/45 domains in *pv38* region A. Numbering is based on the Sal-I reference sequence. *: p < 0.000, †: p < 0.002.

**Table 5:** McDonald-Kreitman test for evaluating the action of natural selection

		***P. vivax/P. cynomolgi***			***P. vivax/P. knowlesi***		
**Worldwide isolates**							
		**Fixed**	**Polymorphic**	**P**_**N**_**/P**_**S **_**> D**_**N**_**/D**_**S **_**p-values**	**Fixed**	**Polymorphic**	**P**_**N**_**/P**_**S **_**> D**_**N**_**/D**_**S **_**p-values**
*pv12*	**Non-synonymous substitutions**	78.66	4	*0.002*	93.86	4	*0.000*
	**Synonymous substitutions**	190.23	0		340.47	0	
*pv38*	**Non-synonymous substitutions**	85.90	6	*0.004*	85.14	6	*0.003*
	**Synonymous substitutions**	257.31	3		265.94	3	
**Colombian population**							
*pv12*	**Non-synonymous substitutions**	93.05	1	0.146	115.54	1	0.083
	**Synonymous substitutions**	197.20	0		347.80	0	
*pv38*	**Non-synonymous substitutions**	89.22	5	*0.023*	88.50	5	*0.016*
	**Synonymous substitutions**	248.66	3		264.90	3	

The McDonald-Kreitman test was done using sequences obtained from databases (worldwide isolates) together with Colombian ones, and just with those obtained in the Colombian population. The interspecies divergence data were obtained from comparing *Plasmodium vivax* sequences with two related species: *Plasmodium cynomolgi* and *Plasmodium knowlesi*. Significant values are shown in italics.

### Genetic diversity in *pv38*

Only 46 out of 70 samples could be amplified for the *pv38* gene, giving a 1,121 bp fragment (South-west n = 6; South-east: n = 13; Midwest: n = 4; North-west: n = 23). The 46 sequences obtained from Colombian isolates were compared to and analysed regarding reference sequences obtained from different regions of the world [43,44]. Colombian sequences that have a different haplotype to that of previously reported ones can be found in GenBank (accession numbers KF667330-KF667340).

Nine SNPs were observed in the *pv38* gene (Figure 1B), most of which were no-synonymous (nucleotides: 88 (R30S), 206/207 (A69V), 209 (R70L), 524/525 (T175N), 880 (M294L), and 998 (S333N)), similar to that found in *Pf38*[73]. Positions 525 and 969 produced synonymous substitutions (a change in protein sequence was generated when the substitution in position 525 was accompanied with another one in position 524). The parasite population in Colombia has eight of these nine SNPs, all being informative-parsimonious sites. Similar to that reported for its orthologue in *P. falciparum*[73], most substitutions were found in the gene’s 5′ region.

Seventeen haplotypes were identified from alignment (including sequences from different regions of the world) (Figure 1B), 14 of which were found in Colombia’s parasite population at different frequencies: 11% haplotype 1, 4% haplotype 5, 2% haplotype 6, 20% haplotype 7, 15% haplotype 8, 7% haplotype 9, 2% haplotype 10, 4% haplotype 11, 13% haplotype 12, 4% haplotype 13, 2% haplotype 14, 4% haplotype 15, 7% haplotype 16, and 4% haplotype 17. Most haplotypes were found in intermediate frequencies per year (2007 n = 6; 2008 n = 6; 2009 n = 8; 2010 n = 26) and none exceeded 50% (Figure 2B). The absence of some haplotypes in determined years, or in some locations, could not just have been due to the low frequency which they might have had but also to the difference in the number of samples for each year (n = 6 in 2007, n = 6 in 2008, n = 8 in 2009 and n = 26 in 2010) or because American *P. vivax* populations appear to be structured and therefore several privative haplotypes might be found [67].

π in this gene was 0.0026 ± 0.0002 worldwide and 0.0024 ± 0.0002 in the Colombian population (Table 1), this being 1.3 times lower than that for its orthologue in *P. falciparum* (π = 0.0034) [68,73] showing that the *pv38* gene had low diversity, at least in the two main species affecting human beings.

### Deviation from the neutral model of molecular evolution in *pv38*

Tajima’s D, Fu and Li’s D* and F*, Fay and Wu’s H and Fu’s Fs neutrality tests did not reveal statistically significant values (Table 2), suggesting that the gene might follow the neutral evolution model. However, the presence of 14 haplotypes and 0.890 ± 0.02 haplotype diversity in the Colombian population was greater than that expected under neutrality according to K-test and H-test results (Table 2). This suggested balanced ancestral polymorphism [48], being similar to that reported for the *P. falciparum p38* gene which showed evidence of balanced selection in 5’ region [73].

### Natural selection in *pv38*

A modified Nei Gojobori method was used for calculating d_N_ and d_S_ rates for showing some type of selection in the *pv38* gene. Similar to that used regarding *pv12*, the *pv38* gene was divided into two regions: region A, covering position 1-459 (amino acids 1-153) and region B, nucleotides 460-1,065 (amino acids 154-355 including the s48/45 domain). There were more d_N_ substitutions in region A than d_S_ substitutions, whilst there were more d_S_ substitutions in region B than d_N_ ones, even though no significant values were observed (Table 4 and Additional file 2). Selection tests by codon revealed positive selection in codon 70 and negative selection in codons 175 and 323, suggesting that the gene was influenced by selection. When the long-term effect of natural selection was explored by comparing divergence rates (K_S_ and K_N_), *pv38* had a higher statistically significant K_S_ rate than K_N_ (Table 4), revealing ω values below 1 throughout the gene (Figure 3), suggesting divergence by negative selection.

The McDonald-Kreitman test revealed statistically significant values (Table 4), when intraspecific polymorphism and interspecific divergence was compared, showing P_N_/P_S_ > D_N_/D_S_ (p < 0.02). This result could have been the result of either a negative selection or a balanced selection [61,74]. K-test and H-test results (Table 2) and the presence of different haplotypes at intermediate frequencies (Figure 2B) suggested that it is most probable that *pv38* was influenced by balanced selection, similar to that reported for *P. falciparum*[73]. Such selection seemed to be domain specific. Significant values were observed for region A (p = 0.014) when intraspecific polymorphism and interspecific divergence was calculated in each region (Additional file 3), this being where most of the substitutions found became accumulated, whilst neutrality could not be ruled out for region B (p = 0.1). Functional/structural constraint due to the presence of an s48/45 domain was also probable for *pv38*, given this region’s low diversity, two negatively selected sites and a statistically significant K_S_ > K_N_.

### Linkage disequilibrium (LD) and recombination

Several statistics were calculated for determining possible associations between polymorphisms and/or the presence of recombination in *pv38*. Z_nS_ did not reveal statistically significant values, indicating that *pv38* polymorphisms were not associated. Lineal regression between linkage disequilibrium (LD) and nucleotide distance revealed a reduction in LD as nucleotide distance increased, indicating that intragenic recombination might have led to new variations being produced.

The ZZ statistic was calculated to confirm whether recombination affected *pv38* evolution, showing no significant values (Table 2); however, 2 RM (minimum recombination events) were found. The GARD method (in Datamonkey web server) gave a recombination breakpoint in position 524. Prior studies have suggested that new haplotypes could be produced through recombination in spite of functional constraints [73]. Intragenic recombination could thus be one of the factors promoting diversity in the *pv38* gene. Crosslinking during recombination could produce new combinations between the gene’s 5′ (region A) and 3′ region (region B) as the breakpoint found in this gene was located upstream of the region encoding the s48/45 domain (region B). As only one polymorphic site was found in *pv12*, the aforementioned tests were not carried for this gene.

### *pv12* and *pv38* should be considered for an antimalarial vaccine

The lack of a totally effective vaccine against human malarial parasites is at least partly due to high genetic diversity found in proteins involved in red blood cell invasion. These molecules’ constant exposure to the host’s immune system allows the fixation of mutations generating an adaptive advantage preventing their recognition. Antigens such as *pvmsp-1*, *pvdbp*, *pvmsp-3*α, *pvmsp-5*, *pvmsp-7C*, *pvmsp-7H*, *pvmsp-7I* and *pvama-1* have shown high genetic diversity which appears to be maintained by positive-balancing selection [10-15,75-78]; however, other antigens are highly conserved despite being exposed to the host’s immune system. Surface antigens such as *pvmsp-4*, *pvmsp-7A*, *pvmsp-7 K*, *pvmsp-8*, *pvmsp-10*, *pv230* or others in the rhoptries (*pvrap-1* and *pvrap-2*) appear to evolve more slowly due to a possible functional constraint in their encoded proteins [70,71,79-82]. Thus, most mutations have become eliminated from the population, maintaining a conserved protein structure, even throughout these parasites’ evolutionary history [70,71]. The latter behaviour seems to have been directing *pv12* and *pv38* evolution, highlighting high conservation at both intra- and inter-species level due to the influence of negative selection exerted on s48/45 domains which are important for red blood cell recognition [30]. Although antigens having low genetic diversity are usually not immunogenic [83] nor do they induce protection-inducing responses [84], some limited polymorphism antigens have been shown to be able to induce immunogenicity and protection [85]. Therefore, *pv12* and *pv38* (or their s48/45 domains) should be evaluated regarding vaccine development because immune responses against 6-Cys family antigens appear to be directed against structural epitopes in s48/45 domains [86-88], blocking such domains should prevent invasion [30,88] and being highly conserved and having a functional constraint, allele-specific immune responses are thus avoided.

## Conclusions

The *p12* and *p38* genes in *P. vivax* were seen to have low genetic diversity; the regions encoding the s48/45 domains seemed to be functionally or structurally constrained. Several members of the 6-Cys family are found on the surface of malaria parasites in every stage [28,36-39,69] and some of them (e g, P48/45, P230) are considered to be promising (transmission-blocking) vaccine candidates [36,37,87]. Epitopes identified by monoclonal antibodies against this type of protein are structural and have been localized within s48/45 domains [86,87] which seem to be involved in host-pathogen interaction [30,72]. Since *pv12* and *pv38* share structural characteristics with members of the 6-Cys family, added to their antigenic characteristics [38-40] and the low genetic diversity found in this study, the proteins encoded by these genes or their functionally/structurally constrained (conserved) regions could be born in mind when designing a multistage, multi-antigen subunit-based anti-malarial vaccine.

## Competing interests

The authors declare that they have no competing interests.

## Authors’ contributions

JF-R devised the study, participated in designing it, performed the experiments, made the population genetics analysis and wrote the manuscript. DG-O devised and designed the study, helped perform the experiments, carried out the population genetics analysis and wrote the manuscript. MAP devised and coordinated the study, and helped to write the manuscript. All the authors have read and approved the final manuscript.

## Supplementary Material

Additional file 1 ***pv12 *** **and *pv38 *** **haplotypes distribution in the Colombian population.** Haplotype distribution found in *pv12* (A) and *pv38* (B) from 2007 to 2010. Click here for file

Additional file 2 **Synonymous substitution per synonymous site rate (d**_**S**_**) and non-synonymous substitution per non-synonymous site rate (d**_**N**_**) in s48/45 domains from *pv12 *** **and *pv38 *** **genes.** No statistically significant differences were found by codon-based Z-test or Fisher’s exact tests. se: Standard error. *pv12* s48/45 domain in region A: nucleotides 82-471; *pv12* s48/45 domain in region B: nucleotides 589-906; *pv38* s48/45 domain in region B: nucleotides 481-852 -: There is no s48/45 domain in the *pv38* region. Numbering is based on the Sal-I reference sequence. Click here for file

Additional file 3 **McDonald-Kreitman test for evaluating the action of natural selection in *pv12 *** **and *pv38 *** **gene regions A and B.** The McDonald-Kreitman test was done using sequences obtained from databases (worldwide isolates) together with Colombian ones, and just with those obtained in the Colombian population. The interspecies divergence data was obtained from comparing *Plasmodium vivax* sequences with two related species: *Plasmodium cynomolgi* and *Plasmodium knowlesi*. Significant values are underlined. *pv12*: region A, nucleotides 1-546 and region B, nucleotides 547-1,095. *pv38*: region A, nucleotides 1-459 and region B, nucleotides 460-1,065. Click here for file
