# Supplementary material for: Low genetic diversity and functional constraint in loci encoding Plasmodium vivax P12 and P38 proteins in the Colombian population
Source: Malar J. 2014 Feb 18;13:58. doi: 10.1186/1475-2875-13-58 (PMC3930544; doi:10.1186/1475-2875-13-58)
Supplement: Additional file 1 — pv12 and pv38 haplotypes distribution in the Colombian population. Haplotype distribution found in pv12 (A) and pv38 (B) from 2007 to 2010. [file 1475-2875-13-58-S1.doc]

**
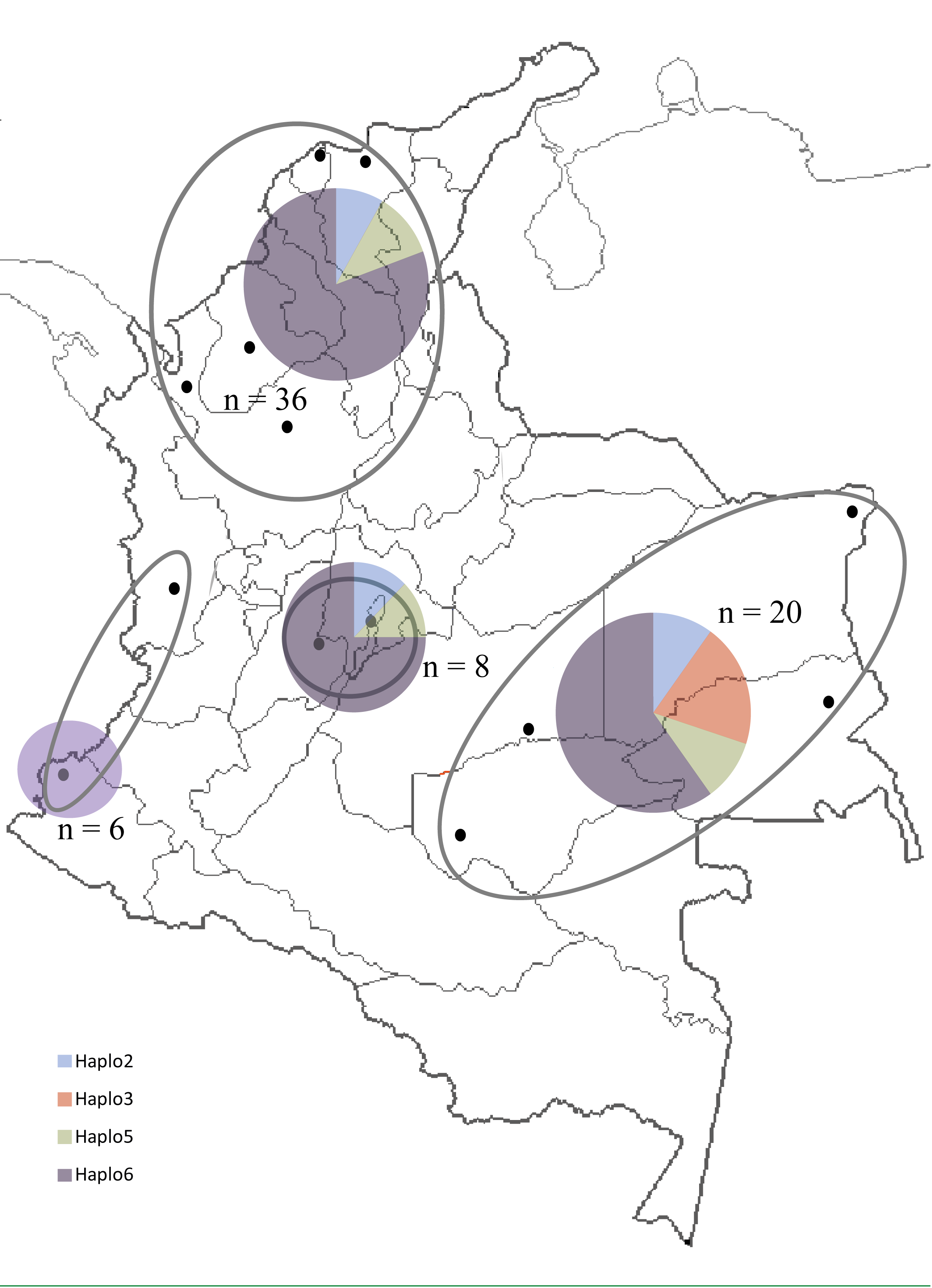
**

**Additional file 1** **A.** haplotype distribution found in *pv12* from 2007 to 2010

North-west: Haplo_2 (10%) Haplo_5 (10%) Haplo_6 (80%)

South-east: Haplo_2 (11%) Haplo_3 (21%) Haplo_5 (11%) Haplo_6 (58%)

Midwest Haplo_2 (13%) Haplo_5 (13%) Haplo_6 (75%)

South-west: Haplo_6 (100%)

*
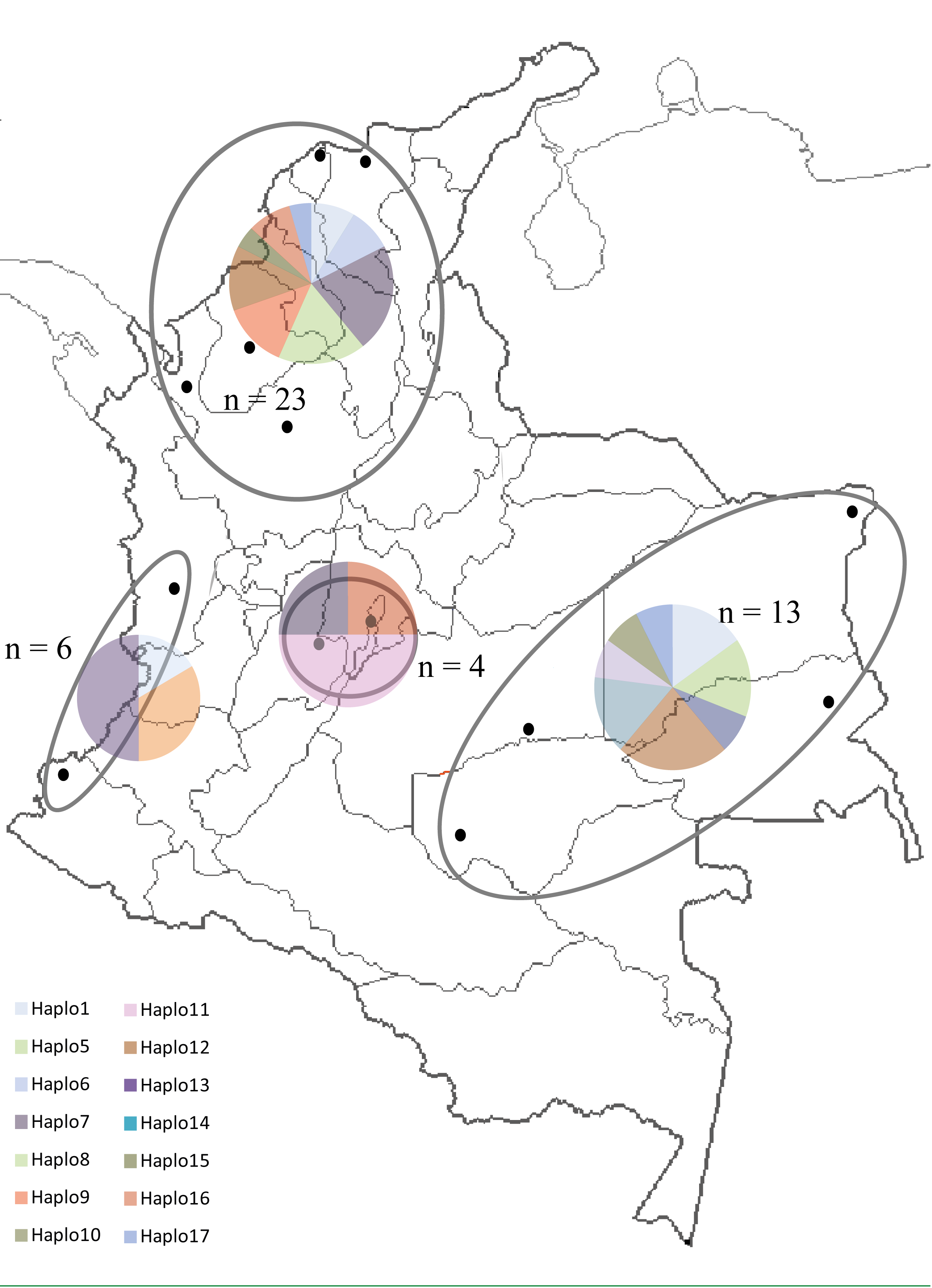
*

**B.** haplotype distribution found in *pv38* from 2007 to 2010

North-west: Haplo_1 (0,1%) Haplo_6 (15%) Haplo_7 (22%) Haplo_8 (17%) Haplo_9 (13%) Haplo_12 (13%) Haplo_15 (0,4%) Haplo_16 (0,9%) Haplo_17 (0,8%)

South-east: Haplo_1 (15%) Haplo_8 (15%) Haplo_10 (0,8%) Haplo_12 (23%) Haplo_13 (15%) Haplo_14 (0,8%) Haplo_15 (0,8%) Haplo_17 (0,8%)

Midwest Haplo_7 (25%) Haplo_11 (50%) Haplo_16 (25%)

South-west: Haplo_1 (17%) Haplo_5 (33%) Haplo_7 (50%)
