# Supplementary material for: Low genetic diversity and functional constraint in loci encoding Plasmodium vivax P12 and P38 proteins in the Colombian population
Source: Malar J. 2014 Feb 18;13:58. doi: 10.1186/1475-2875-13-58 (PMC3930544; doi:10.1186/1475-2875-13-58)
Supplement: Additional file 2 — Synonymous substitution per synonymous site rate (d S ) and non-synonymous substitution per non-synonymous site rate (d N ) in s48/45 domains from pv12 and pv38 genes. No statistically significant differences were found by codon-based Z-test or Fisher’s exact tests. se: Standard error. pv12 s48/45 domain in region A: nucleotides 82-471; pv12 s48/45 domain in region B: nucleotides 589-906; pv38 s48/45 domain in region B: nucleotides 481-852 -: There is no s48/45 domain in the pv38 region. Numbering is based on the Sal-I reference sequence. [file 1475-2875-13-58-S2.docx]

**Additional file 2 Synonymous substitution per synonymous site rate (d_S_) and non-synonymous substitution per non-synonymous site rate (d_N_) in s48/45 domains from *pv12* and *pv38* genes.**

| ***Plasmodium vivax*** | | | | | |
| --- | --- | --- | --- | --- | --- |
| **n** | **Gene** | **s48/45 domain in region A** | | **s48/45 domain in region B** | |
| **Worldwide isolates** | | **d_S_ (se)** | **d_N_ (se)** | **d_S_ (se)** | **d_N_ (se)** |
| 76 | ***pv12*** | 0.0000 (0.0000) | 0.0013 (0.0012) | 0.0000 (0.0000) | 0.0001 (0.0001) |
| 53 | ***pv38*** | - | | 0.0047 (0.0046) | 0.0010 (0.0009) |
| **Colombian isolates** | |  |  |  |  |
| 70 | ***pv12*** | 0.0000 (0.0000) | 0.0010 (0.0010) | 0.0000 (0.0000) | 0.0000 (0.0000) |
| 46 | ***pv38*** | - | | 0.0041 (0.0041) | 0.0006 (0.0006) |

No statistically significant differences were found by codon-based Z-test or Fisher’s exact tests. se: Standard error. *pv12* s48/45 domain in region A: nucleotides 82-471; *pv12* s48/45 domain in region B: nucleotides 589-906; *pv38* s48/45 domain in region B: nucleotides 481-852 -: There is no s48/45 domain in the *pv38* region. Numbering is based on the Sal-I reference sequence.
