# Supplementary material for: Low genetic diversity and functional constraint in loci encoding Plasmodium vivax P12 and P38 proteins in the Colombian population
Source: Malar J. 2014 Feb 18;13:58. doi: 10.1186/1475-2875-13-58 (PMC3930544; doi:10.1186/1475-2875-13-58)
Supplement: Additional file 3 — McDonald-Kreitman test for evaluating the action of natural selection in pv12 and pv38 gene regions A and B. The McDonald-Kreitman test was done using sequences obtained from databases (worldwide isolates) together with Colombian ones, and just with those obtained in the Colombian population. The interspecies divergence data was obtained from comparing Plasmodium vivax sequences with two related species: Plasmodium cynomolgi and Plasmodium knowlesi. Significant values are underlined. pv12: region A, nucleotides 1-546 and region B, nucleotides 547-1,095. pv38: region A, nucleotides 1-459 and region B, nucleotides 460-1,065. [file 1475-2875-13-58-S3.docx]

**Additional file 3 McDonald-Kreitman test for evaluating the action of natural selection in *pv12* and *pv38* gene regions A and B.**

|  |  | | | | | | ***Plasmodium vivax/Plasmodium cynomolgi*** | | | | | | | ***Plasmodium vivax/ Plasmodium knowlesi*** | | | | |  |  |
| --- | --- | --- | --- | --- | --- | --- | --- | --- | --- | --- | --- | --- | --- | --- | --- | --- | --- | --- | --- | --- |
| Region A | **Worldwide isolates** | | | | | | | | | | | | | | | | | |  |  |
|  |  | | |  | **Fixed** | | | **Polymorphic** | | **P_N_/P_S_ < D_N_/D_S_**  **p-values** | | **Fixed** | | | **Polymorphic** | | **P_N_/P_S_ < D_N_/D_S_**  **p-values** | |  |  |
|  | *pv12* | | | **Non-synonymous substitutions** | 38.9 | | | 2 | | 0.037 | | 47.21 | | | 2 | | 0.001 | |  |  |
|  |  |  |  | **Synonymous substitutions** | 87.08 | | | 0 | |  |  | 242.59 | | | 0 | |  |  |  |  |
|  | *pv38* | | | **Non-synonymous substitutions** | 38.78 | | | 3 | | 0.014 | | 31.07 | | | 3 | | 0.000 | |  |  |
|  |  |  |  | **Synonymous substitutions** | 133.51 | | | 1 | |  |  | 213.33 | | | 1 | |  |  |  |  |
|  | **Colombian population** | | | | | | | | | | | | | | | | | |  |  |
|  | *pv12* | | | **Non-synonymous substitutions** | 46.59 | | | 1 | | 0.164 | | 57.37 | | | 1 | | 0.080 | |  |  |
|  |  |  |  | **Synonymous substitutions** | 91.47 | | | 0 | |  |  | 178.03 | | | 0 | |  |  |  |  |
|  | *pv38* | | | **Non-synonymous substitutions** | 38.78 | | | 3 | | 0.014 | | 31.07 | | | 3 | | 0.000 | |  |  |
|  |  |  |  | **Synonymous substitutions** | 133.52 | | | 1 | |  |  | 213.46 | | | 1 | |  |  |  |  |
|  | | |  |  | | | ***Plasmodium vivax/Plasmodium cynomolgi*** | | | | | | | ***Plasmodium vivax/ Plasmodium knowlesi*** | | | | | | |
| Region B | | | **Worldwide isolates** | | | | | | | | | | | | | | | | | |
|  |  |  |  |  | | | **Fixed** | | | **Polymorphic** | | **P_N_/P_S_ < D_N_/D_S_**  **p-values** | | **Fixed** | | | **Polymorphic** | | **P_N_/P_S_ < D_N_/D_S_**  **p-values** | |
|  |  |  | *pv12* | **Non-synonymous substitutions** | | | 39.77 | | | 2 | | 0.025 | | 46.67 | | | 2 | | 0.013 | |
|  |  |  |  | **Synonymous substitutions** | | | 103.18 | | | 0 | |  |  | 148.35 | | | 0 | |  |  |
|  |  |  | *pv38* | **Non-synonymous substitutions** | | | 47.12 | | | 3 | | 0.109 | | 54.10 | | | 3 | | 0.182 | |
|  |  |  |  | **Synonymous substitutions** | | | 125.50 | | | 2 | |  |  | 116.77 | | | 2 | |  |  |
|  |  |  | **Colombian population** | | | | | | | | | | | | | | | | | |
|  |  |  | *pv12* | **Non-synonymous substitutions** | | | 46.46 | | | 0 | | ND | | 58.17 | | | 0 | | ND | |
|  |  |  |  | **Synonymous substitutions** | | | 105.80 | | | 0 | |  |  | 172.20 | | | 0 | |  |  |
|  |  |  | *pv38* | **Non-synonymous substitutions** | | | 50.44 | | | 2 | | 0.381 | | 57.47 | | | 2 | | 0.461 | |
|  |  |  |  | **Synonymous substitutions** | | | 119.42 | | | 2 | |  |  | 119.35 | | | 2 | |  |  |

The McDonald-Kreitman test was done using sequences obtained from databases (worldwide isolates) together with Colombian ones, and just with those obtained in the Colombian population. The interspecies divergence data was obtained from comparing *Plasmodium vivax* sequences with two related species: *Plasmodium cynomolgi* and *Plasmodium knowlesi*. Significant values are underlined. *pv12*: region A, nucleotides 1-546 and region B, nucleotides 547-1,095. *pv38*: region A, nucleotides 1-459 and region B, nucleotides 460-1,065.
